# Late‐onset Pompe disease with a novel mutation and a rare phenotype: A case report

**DOI:** 10.1111/cns.13903

**Published:** 2022-07-07

**Authors:** Xiaoli Si, Ruoxia Zhang, Shengqiang Yan, Guohua Zhao, Xinzhen Yin, Baorong Zhang

**Affiliations:** ^1^ Department of Neurology, The Fourth Affiliated Hospital, International Institutes of Medicine Zhejiang University School of Medicine Zhejiang China; ^2^ Department of Neurology, Second Affiliated Hospital, School of Medicine Zhejiang University Zhejiang China

Dear Editor，

Pompe disease (OMIM 232300) is an autosomal recessive metabolic disorder caused by the deficiency of the acid alpha‐glucosidase (GAA), a lysosome enzyme that hydrolyses glycogen to glucose and is encoded by the *GAA* gene.^1^ Pompe disease is classified into two categories: infantile‐onset Pompe disease (IOPD) and late‐onset Pompe disease (LOPD). IOPD is characterized by onset before 12 months with cardiomyopathy, failure to thrive, and respiratory dysfunction. LOPD refers to the remaining patients with Pompe disease onset less than 12 months without cardiomyopathy or onset after 12 months. The typical clinical manifestations of LOPD are progressive proximal muscle weakness, exercise intolerance, and exertional dyspnea.^2^ *GAA* gene is over 28Kb long and is located at chromosome 17q25.2‐q25.3. Over 679 variants have been described in the *GAA* gene, most of which are pathogenic. In Asia, the most common variants were c.1935C>A and c.2238G>C in Chinese, and c.1857C>G and c.1316T>A in Korean.^3^ Here, we reported a patient affected by LOPD with a “stroke onset.” He has compound heterozygous missense mutations one of which represents a new mutation in the GAA. This case may expand the clinical and gene spectrum of LOPD.

A 39‐year‐old man complained of continuously double vision with dizziness for 4 days. He had a history of hypertension and gout, and was treated with extracorporeal lithotripsy 6 years ago for “kidney stones.” He grew up with average sports performance and was easily fatigued. His family members are in good health. He had limited abduction of both eyes, mild horizontal nystagmus to the left, diplopia to the left, and decreased head‐raising muscle strength. Physical examination of the motor system showed a thin build with mild scoliosis, normal muscle tone, grade 5 muscle strength in both upper limbs, grade 4 proximal muscle strength (similar to usual), and grade 5 distal muscle strength in both lower limbs. Bilateral finger nose, coordinated rotation, and accurate heel–knee tibial tests were stable and accurate; Romberg's sign (−), but difficulty walking in a straight line. Tendon reflexes suggested symmetrical tendon reflexes in both upper limbs (++), bilateral knee reflexes (+), and bilateral ankle reflexes were absent. Bilateral Babinski's sign and Chaddock's sign were positive. Sensory system and autonomic function tests were normal. Laboratory examination revealed a positive result for creatine kinase 458 U/L, lactate dehydrogenase 259 U/L, and blood uric acid 610 μmol/L. GAA enzyme activity was 0.28 μmol/L/h (1.46–20.34)]. Sanger sequencing analysis revealed two compound heterozygous missense mutations in GAA: c.2238G>C (p.W746C; from his father) and c.2296T>C (p.Y766H; from his mother; Panel, arrows; Figure 1). Magnetic resonance imaging (MRI) revealed an acute lacunar infarct on the left side of the pontine brain and lacunar foci on the right side of the pontine brain (Figure 2A). Computed tomography angiography (CTA) suggested bilateral intracranial stenosis of the vertebral artery, with the left side predominant (the high likelihood of a dissecting aneurysm; Figure 2B). Ambulatory blood pressure, ambulatory electrocardiogram, transthoracic echocardiogram, arteriovenous ultrasound of the extremities, and bilateral cervical vascular ultrasound did not show any significant abnormalities. He was diagnosed with LOPD and brainstem infarction, and cerebral vessel involvement was underrecognized complication of this disease. He was treated with enzyme replacement therapy (ERT; alglucosidase alfa), placed on a low‐salt, low‐purine diet, and treated with aspirin (100 mg/day), clopidogrel (75 mg/day), and atorvastatin (20 mg/day). After 6 months of follow‐up, he was free of visual double vision with normal eye movement in all directions.

**FIGURE 1:**
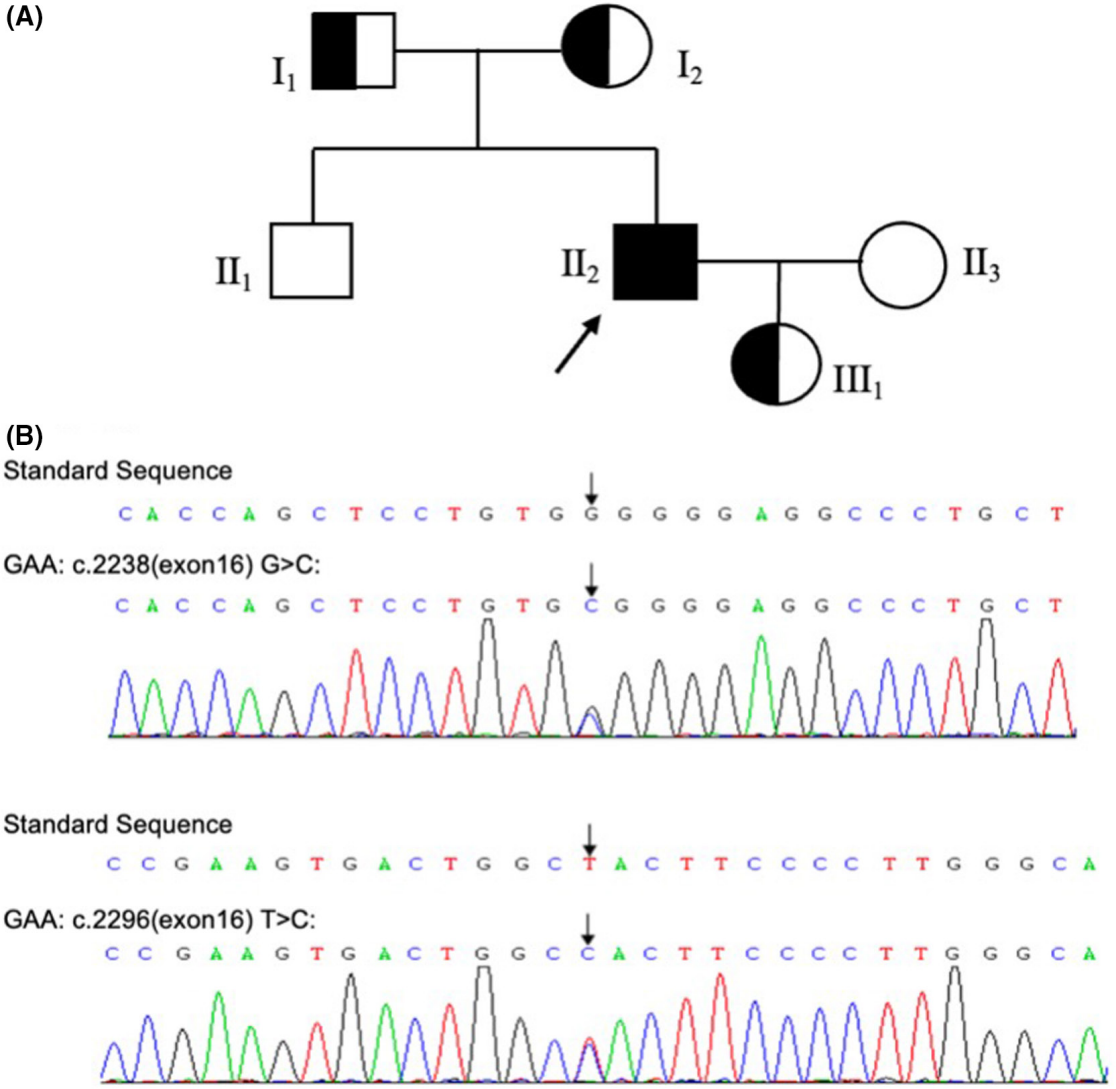
Genetic pedigree of the family and molecular genetic analysis. (A) Genetic pedigree of the family. II‐2 is the proband. (B) The genotype of the patient

**FIGURE 2:**
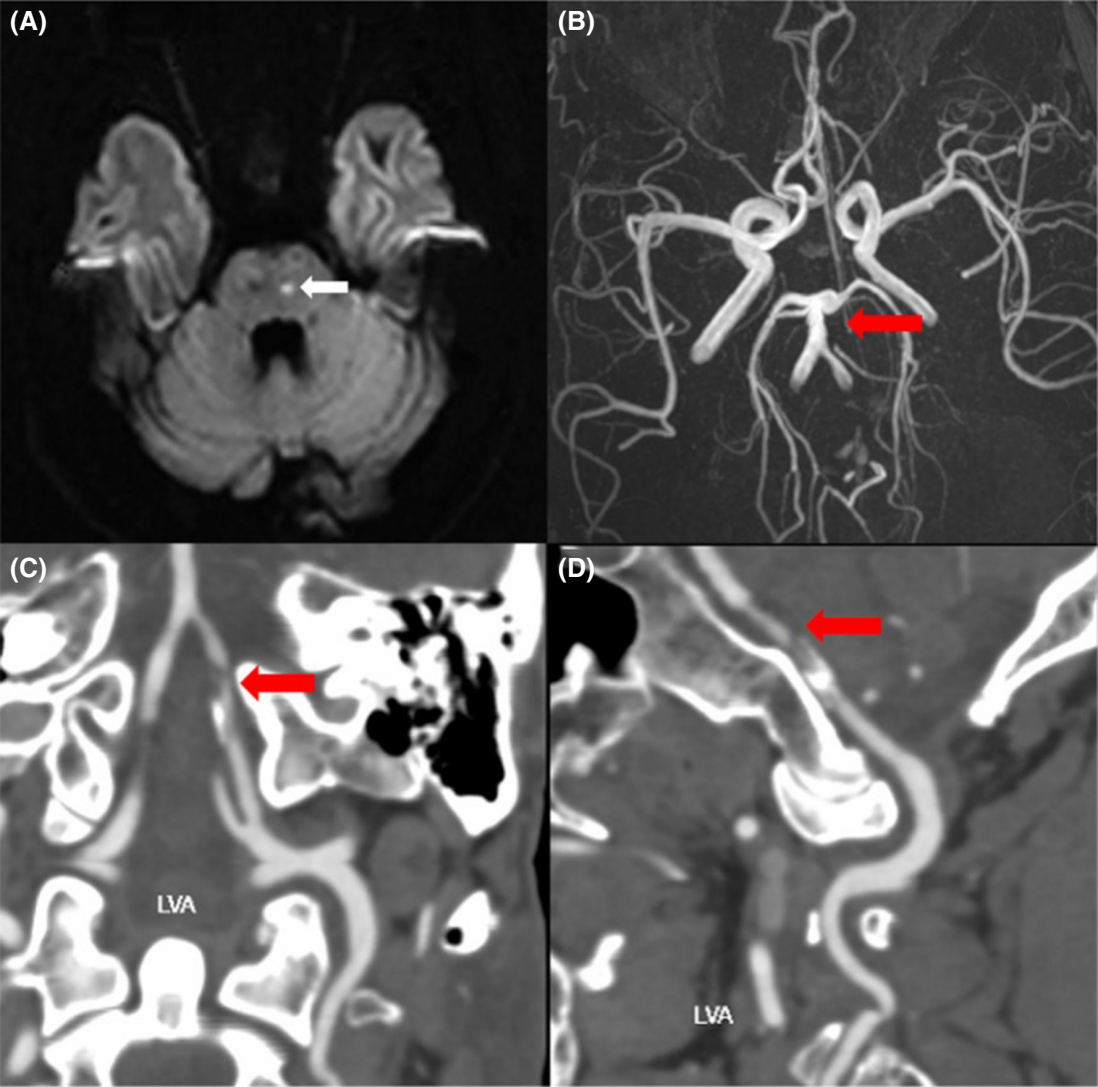
Brain imaging presentations. (A) MRI DWI revealed an acute lacunar infarct on the left side of the pontine brain and lacunar foci on the right side of the pontine brain (white arrow). (B) Brain MRA and (C‐D) CTA showed bilateral vertebral artery stenosis, with the left side predominant (the high likelihood of a dissecting aneurysm; red arrow). Abbreviation: CTA, computed tomography angiography; DWI, diffusion‐weighted imaging; MRA, MR angiography; MRI, magnetic resonance imaging

The estimated birth incidence of LOPD in Chinese was approximately 1/50,000 or more. LOPD tends to have an earlier symptom onset and faster disease progression in Chinese, which may become chair‐bound and require assisted ventilation, even develop into type 2 respiratory failure requiring ventilator support.^4^ However, the diagnosis of LOPD was difficult due to unawareness and diverse disease patterns with a mean diagnostic delay of 7 years. ERT has already shown positive effects in Pompe disease.^5^ Therefore, early diagnosis and appropriate treatment would be necessary to improve the prognosis of LOPD. Our patient demonstrated key diagnostic features that lead the clinician to consider LOPD, which presents a decreased proximal segment muscle strength with increased creatine kinase and bent spine syndrome. Although a rare incidence of cerebral vascular complications in LOPD, artery aneurysms, dilative arteriopathy, and cerebral microbleeds have already been reported, which may be an underrecognized complication.^6^ Several cohort studies show that LOPD has an increased incidence of intracranial aneurysms compared with the normal population.^7^ Our patient presented with posterior circulation infarction and a possibility of an intracranial aneurysm. Therefore, we proposed a differential diagnosis of LOPD and measure GAA enzyme activity in young patients who have an unexplained basilar aneurysm or dilative arteriopathy of intracranial vertebral and basilar arteries. Furthermore, our patient was diagnosed with “kidney stones,” which may be underestimated as a kidney infarct caused by arteriopathy.

Our patient carries a novel compound heterozygous mutation. The missense mutation c.2238G>C, which is located in exon 16, has first been identified in six juvenile‐onset Chinese Pompe disease patients and was found to be the most common mutation in 14 LOPD patients in eastern China.^8^ Jia et al. verified the function of c.2238G>C leading to deficiency in GAA protein expression and enzyme activity from two Pompe siblings both with cerebral infarction.^9^ To our interest, according to HGMD (http://www.hgmd.cf.ac.uk), c.2296T>C has not been previously observed in Pompe disease patients and was predicted as “probably damaging” in the GAA. The mutation of c.2296T>C (p.Y776H) and the mutation of c.2297A>C (p.Y776S) affect the same amino acid residue Y766, which is located in the glycoside hydrolase domain. The mutation p.Y776H would likely disrupt normal protein configuration and impair the function of GAA protein glycoside hydrolase.^10^ Furthermore, the mutations of c.2296T>C and c.2238G>C both locate in exon 16, which may indicate the potential genetic risk of the ischemic stroke onset. Therefore, the mutations identified in this case may be associated with the vascular phenotype of Pompe disease. We will follow up with this patient to investigate whether ERT would influence vascular complications and avoid recurrent cerebrovascular accidents.

## AUTHOR CONTRIBUTIONS

S.XL. and Z.RX. wrote the manuscript. Y.XZ. and Y.SQ. were the physicians who treated the patient. Z.BR. and Z.GH. provided professional comments to the manuscript. All authors have read and approved the final manuscript.

## CONFLICT OF INTEREST

The authors declare that the research was conducted in the absence of any commercial or financial relationships, which could be construed as a potential conflict of interest.

## Data Availability

The data that support the findings of this study are available from the corresponding author upon reasonable request.
